# Review articles (Meta-Analyses) effects of walking on cognitive function in individuals with mild cognitive impairment: a systematic review and meta-analysis

**DOI:** 10.1186/s12877-023-04235-z

**Published:** 2023-08-21

**Authors:** Jia-Chi Lin, I-Hsuan Chen, Fang-Yu Cheng

**Affiliations:** 1 MacKay Medical College, Institute of Long-Term Care, No.46, Sec. 3, Zhongzheng Rd, Sanzhi Dist, New Taipei City, 252 Taiwan; 2 Department of Physical Therapy, Fooyin University, Kaohsiung City, Taiwan

**Keywords:** Mild cognitive impairment, Walking, Cognition, Endurance, Meta-analysis

## Abstract

**Background:**

Mild cognitive impairment (MCI) is the stage between the expected cognitive decline of normal aging and the more serious decline of dementia. Previous studies have shown that regular exercise can improve cognition and physical performance in older adults. Walking is a low-technology and low-cost exercise that has been proven to improve cognition and mobility in healthy elderly individuals. However, no systematic review or meta-analysis has explored whether walking can improve cognitive function in older adults with MCI. This study aimed to explore the effects of walking interventions on cognitive functions in individuals with MCI.

**Methods:**

In accordance with the PRISMA guidelines, MEDLINE, PubMed, SPORTDiscus, Cochrane Central Register of Controlled Trials, CINAHL, Web of Science, Airiti Library, and the National Digital Library of Theses and Dissertations in Taiwan were searched from inception to July 2023. Independent reviewers selected randomized clinical trials (RCT) that compared the effects of walking with no intervention or other exercises in individuals with MCI. The primary outcomes were cognitive functions, and the secondary outcome was walking endurance. Three reviewers independently conducted data extraction. The risk of bias was assessed using the Revised Cochrane Risk of Bias assessment tool.

**Results:**

Fourteen RCTs were included in this review. The quality of evidence in these studies was rated as good to excellent. The results of the meta-analysis showed that the individuals with MCI had no significant improvement in cognitive function but had significant improvement in the 6-min walk test (Mean Difference=23.70, *p*=0.008) after walking interventions compared to no intervention or other exercises.

**Conclusion:**

Walking intervention has no significant improvement on cognitive functions in older adults with MCI. However, walking induces beneficial effects on aerobic capacity.

**Trial registration:**

This systematic review has the registration number CRD42021283753 on PROSPERO.

**Supplementary Information:**

The online version contains supplementary material available at 10.1186/s12877-023-04235-z.

## Introduction

Mild cognitive impairment (MCI) is a clinical condition between normal aging cognition and dementia; the degree of cognitive impairment is higher than average for the same age but not enough to seriously affect daily function [1, 2]. The incidence of MCI in people older than 65 years is approximately 3% to 22% [3–5]. The rate of MCI progression to dementia is 5% to 15% per year, while the incidence of MCI in the general population is 1% to 2% annually [6–8]. The cognitive domains of MCI decline, including learning and memory, social functions, language, visuospatial functions, complex attention, and executive functions, may all be affected [9]. MCI can also cause reduced postural stability and slow walking speed. A previous study reported that slow walking speed is associated with reduced executive function in MCI [10]. Slow walking speed has also been associated with reduced cognitive processing speed in MCI [11]. The reduction in walking speed is a possible risk factor for MCI in older adults [12]. A study reported that people with MCI walk slowly compared with healthy people [13]. A systematic review and meta-analysis including 36 studies noted a significant relationship between gait speed and cognition in older adults. Compared with normal cognition controls, the walking speed was reduced by 0.11 m/s in participants with MCI, and the walking speed was reduced by 0.2 m/s in those with mild dementia [14]. MCI has an impact on both cognition and walking speed, and it may turn into dementia. Thus, walking is important for MCI and the prevention of dementia.

Walking is the most prevalent type of exercise in the aging population [15]. Past studies have shown that walking has a positive effect on healthy elderly individuals, people at risk of falling, and individuals with stroke, and it is a safe method of intervention [16–18]. Nalbant et al. found that 6 months of supervised walking program decreased body weight and improved performance in the 6-min walk test (6MWT) in older adults [19]. Okubo et al. demonstrated that 12 weeks of walking intervention has similar effects as the balance and strengthening program which contributes to significant improvements in gait speed, dynamic balance, and lower extremity muscle strength [20]. Walking exercise is also a low-technology and low-cost exercise that has been proven to improve cognition in healthy older adults [21]. Colmenares et al. also demonstrated that a 6-month of aerobic walking contributes to white matter plasticity in older adults [22]. Walking is a complex task that requires a delicate balance between various interacting neuronal systems [23]. Walking requires not only automatic motor processes but also advanced balance ability. Cognitive functions such as attention, incoming information processing, and intentional adjustment are also involved [23–25]. The continuous execution of walking over a certain period can improve balance and cognitive ability. A cohort study followed 78430 adults for 6.9 years and found that taking more steps per day may lower the risk of all-cause dementia [26]. As mentioned above, walking is a popular approach to providing physical activity to the general public [27], and it can benefit both physical and cognitive functions in older adults. However, no integration analysis or systematic review study has explored the effect of walking on cognitive function in people with MCI. Therefore, we designed and conducted this systematic review and meta-analysis to explore the effects of walking interventions on cognitive functions in individuals with MCI.

## Methods

### Study design and registration

This systematic review has the registration number CRD42021283753 on PROSPERO. This report follows the Preferred Reporting Items for Systematic Reviews and Meta-Analyses (PRISMA) [28]. The synthesis of the evidence was conducted using the Grading of Recommendations, Assessment, Development and Evaluations (GRADE) approach [29].

### Literature search

We searched MEDLINE, PubMed, SPORTDiscus, Cochrane Central Register of Controlled Trials, CINAHL, Web of Science, Airiti Library, and the National Digital Library of Theses and Dissertations in Taiwan from their inception through July 2023, using a combination of the following MeSH search terms: [(Mild Cognitive Impairment “OR” Cognitive Dysfunction “AND” Walking “OR” Exercise “OR” Brisk Walking)]. The retrieval process was listed in Appendix 1. The reference lists of the included studies or relevant reviews were screened manually for additional studies.

### Inclusion criteria

The trials selected in this review met the following inclusion criteria: (a) study design: randomized controlled trials (RCTs); (b) participants: individuals with MCI by any available diagnostic criteria, such as Petersen criteria, US mental disorders fifth edition of the Diagnostic and Statistical Manual (DSM-5), Mini-Mental State Examination (MMSE) ≥ 24/30, Montreal Cognitive Assessment (MoCA) < 26/30, and other standards and consensus; (c) intervention: walking or brisk walking practiced in the experimental group; (d) control: participants in the control group maintained their usual physical activities or were administered sham exercises (e.g., stretching and balance); (e) language: English or Chinese.

### Outcomes

The primary outcomes were global cognitive function [the MMSE, MoCA] and any specific domains of cognition, including verbal learning [Rey Auditory Verbal Learning Test (RAVLT)], or processing speed [the Digit Symbol Substitution Test (DSST)]. The secondary outcome included walking endurance for the 6MWT. We used the GRADE approach, which analyzes the following domains: limitations, inconsistency, indirectness, imprecision, and publication bias, to evaluate the level of scientific evidence of the meta-analysis [29].

### Study selection

The two reviewers (L-JC and C-FY) independently checked the titles and abstracts of the articles identified in the search. The full text was read if the article could not be identified through the title and abstract. We contacted the corresponding author for information that we needed but did not find in the article. When the two authors had different opinions in the process of research selection, we resolved them through discussion or recourse to the other author (C-IH).

### Study quality

We used the Revised Cochrane Risk of Bias assessment tool (RoB 2) to assess the methodological quality of the trials. The RoB 2 evaluates the quality of the RCT from the following five domains: (a) the randomization process, (b) deviations from the intended intervention, (c) missing outcome data, (d) measurement of outcome, and (e) selection of the reported result. Each domain was graded as high risk, some concerns, or low risk. Each article was evaluated by at least two reviewers (L-JC and C-FY). A third independent reviewer (C-IH) was consulted to resolve disagreements.

### Assessment of publication biases

The funnel plot is used to assess whether there is publication bias in the selected literature for a meta-analysis. When the number of studies in the meta-analysis is less than 10, it is not recommended to use the funnel plot to test for asymmetry [30]. In this study, since the number of studies included for each variable was less than ten, funnel plots were not presented.

### Subgroup analysis

To explore heterogeneity in estimating the difference between the control group (active control group, or usual care), we performed subgroup analyses according to the type of intervention in the control group. We compared the walking exercise effect on processing speed whether the intervention in the control groups varied.

### Sensitivity analysis

We performed a sensitivity analysis to assess how the results of meta-analysis might be affected by a high risk of bias in the measurement of the outcomes (assessors were not blinded) on verbal learning.

### Statistical analysis

The data were analyzed by Review Manager statistical software (RevMan 5.4 version, Cochrane, USA). Following the Cochrane Handbook [31], mean ± standard deviation (SD) for either change from baseline to post-intervention or immediately post-intervention values were combined in a meta-analysis. The RevMan calculator was used to convert standard errors, CIs, or interquartile range to SD where necessary. Continuous measures utilize the mean difference (MD) to measure effect size, which is then presented with a 95% confidence interval. However, when the outcome measures were assessed using different measurement scales, the standardized mean difference (SMD) was utilized to estimate the pooled effect size, which allows for comparison across studies with varying scales.

We conducted a heterogeneity test before the meta-analysis and used the *p value* to check the significance level. Statistically significant differences were defined as *p value*s < 0.05. Heterogeneity among studies was also assessed using the *I*^2^-index statistic. A value of *I*^2^ > 50% accompanied by *p* < 0.1 for the heterogeneity test indicated a moderate to high level of heterogeneity; therefore, a random effect model was used. In contrast, *I*^2^ ≤ 50% accompanied by *p* > 0.1 was expressed as little to moderate heterogeneity using the fixed-effect model.

## Results

### Study identification

**Fig. 1:**
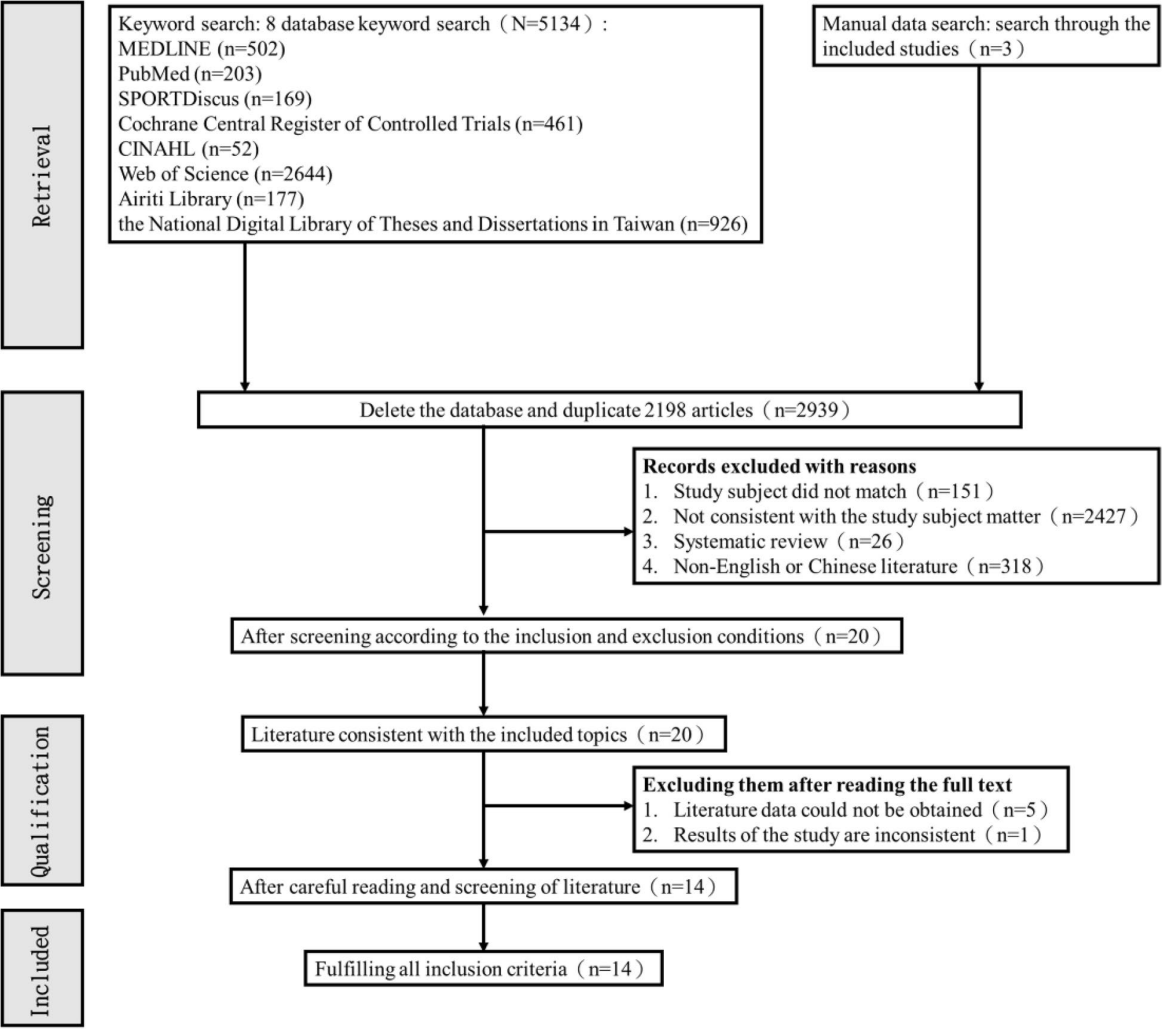
Flow diagram of the included studies

In this study, eight electronic searches and hand searches for additional resources identified 5,134 potential records. After eliminating duplicates and excluding studies based on the eligibility criteria, the number of relevant records was reduced to 20. Among the 20 potentially eligible articles, 5 studies were excluded due to the inability to obtain raw data, we attempted to contact the corresponding authors of these articles via email, but unfortunately, we did not receive any responses, and one study was excluded due to inconsistent outcome measures (economic evaluation). Finally, fourteen studies were included in this systematic review, all fourteen of which were selected for the synthesis of evidence using the GRADE method. The flow chart of study selection and identification is shown in Fig. 1.

### Study characteristics

The characteristics of the 14 included studies are summarized in Table 1. This review included 560 participants with MCI, with 305 in the walking group and 298 in the control group. The participants age ranged from 55 to 80 years old. The studies were published in Canada, America, the Netherlands, and China between 2008 and 2021. The participants were recruited from communities, hospitals, clinics, or elderly care centers [32–45]. The sample size of these studies ranged from 21 to 138. The interventions in the experimental groups were walking or brisk walking, while the comparisons included hand/face exercises, stretching exercises, activities of daily living, social activities, health education, range of motion exercises, balance exercises, usual care, relaxation techniques, flexibility, and postural exercises. Most of the treatment schedules in the included studies were set at 25 to 60 min for each session 2–5 times per week, and the total treatment duration ranged from 24 to 48 weeks. The primary outcome measures included global cognitive function (MMSE and MoCA), verbal learning (RAVLT), and processing speed (DSST). The secondary outcome measure included walking endurance (6MWT).

**Table 1:** Characteristics of the included studies

Study	Mean age	Participants	Interventions	Outcomes
Van Uffelen et al. 2008 [32]	75.10	*n* = 138	walking (moderate-intensity) vs. non-aerobic group exercises (low-intensity) 60 min, 2 times/week, 1 year	MMSE, AVLT, DSST, SCWT-A, BDNF (biospecimen collection)
Barha et al. 2017 [36]	73.84	*n* = 58	walking (HRR:60–70%) vs. usual care and health education (unspecified intensity) 60 min, 3 times/week, 6 months	MMSE, MoCA, ADAS-Cog, TMT B-A, Stroop Test, 6MWT, BDNF (biospecimen collection)
Nagamatsu et al. 2013 [33]	74 .89	*n* = 49	walking (HRR:40–80%) vs. balance and tone (unspecified intensity) 60 min, 2 times/week, 6 months	RAVLT, spatial memory
Dao et al. 2017 [37]	71.73	*n* = 22	walking (HRR:60–70%) vs. usual care and health education (unspecified intensity) 60 min, 3 times/week, 6 months	ADAS-Cog, TMT, Stroop Test, DSST, physiological profile assessment, Amyloid-β imaging (MRI)
Tao et al. 2019 [41]	65.55	*n* = 37	walking (HRR:55–75%) vs. usual care and health education (unspecified intensity) 60 min, 3 times/week, 24 weeks	MoCA, hippocampal volume (MRI)
Ten Brinke et al. 2015 [34]	75.78	*n* = 21	walking (HRR:40–80%) vs. usual care (unspecified intensity) 60 min, 2 times/week, 6 months	RAVLT, hippocampal volume (MRI)
Tarumi et al. 2019 [42]	64.72	*n* = 45	walking (HRR:75–90%) vs. stretching exercises (HRR: < 50%) 25–40 min, 3–5 times/week, 12 months	CVLT-II, D-KEFS, global brain and hippocampal volumes, mean cortical and precuneus Aβ plaque deposition (Amyloid PET)
Tomoto et al. 2021 [43]	64.70	*n* = 37	walking (HRR:75–90%) vs. stretching and toning exercises (HRR: < 50%) 25–40 min, 3–5 times/week, 12 months	CVLT-II, D-KEFS, cerebral blood flow velocity, mean arterial pressure, cerebral vasomotor reactivity
Hsu et al. 2018 [39]	73.03	*n* = 21	walking (HRR:60–70%) vs. usual care and health education (unspecified intensity) 60 min, 3 times/week, 6 month	MMSE, MoCA, 6MWT, cerebral cortex activity
Liu-Ambrose et al. 2016 [35]	74.25	*n* = 58	walking (HRR:60–70%) vs. usual care and health education (unspecified intensity) 60 min, 3 times/week, 6 month	ADAS-Cog, Stroop Test, TMT, 6MWT, EXIT-25, ADCS-ADL
Hsu et al. 2017 [38]	71.1	*n* = 21	walking (HRR:60–70%) vs. usual care and health education (unspecified intensity) 60 min, 3 times/week, 6 month	6MWT, Timed-Up-and-Go Test, Short Physical Performance Battery, physical activities scale for the elderly, cerebral cortex activity
Ten Brinke et al. 2018 [40]	72.39	*n* = 28	walking (HRR:60–70%) vs. usual care and health education (unspecified intensity) 60 min, 3 times/week, 6 month	Stroop Test, TMT, DSST, 6MWT, cerebral cortex activity
Tomoto et al. 2021 [45]	65.45	*n* = 37	walking (HRR:75–90%) vs. stretching and toning exercises (HRR: < 50%) 25–40 min, 3–5 times/week, 12 months	MMSE, CVLT-II, D-KEFS, cardiovascular hemodynamics, cerebrovascular hemodynamics, brain tissue volume, white matter hyperintensity, cardiorespiratory fitness
Liu et al. 2021 [44]	65.15	*n* = 37	walking (HRR:55–75%) vs. original physical activity levels and health education (unspecified intensity)	MoCA, cortical functional connectivity (fMRI)

*Abbreviations: MoCA* Montreal Cognitive Assessment, *ADAS-Cog* Alzheimer's Disease Assessment Scale-Cognitive, *TMT B-A* Trail-Making Tests B-A, *D-KEFS* Delis-Kaplan Executive Function System, *RAVLT* Rey Auditory Verbal Learning Test, *CVLT-II* California Verbal Learning Test-Second Edition, *DSST* Digit Symbol Substitution Test, *HRR* Heart Rate Reserve, *BDNF* Brain-Derived Neurotrophic Factor, *EXIT-25* The Executive Interview, *ADCS-ADL* The Alzheimer's Disease Cooperative Study—Activities of Daily Living Scale

### Study quality

**Fig. 2:**
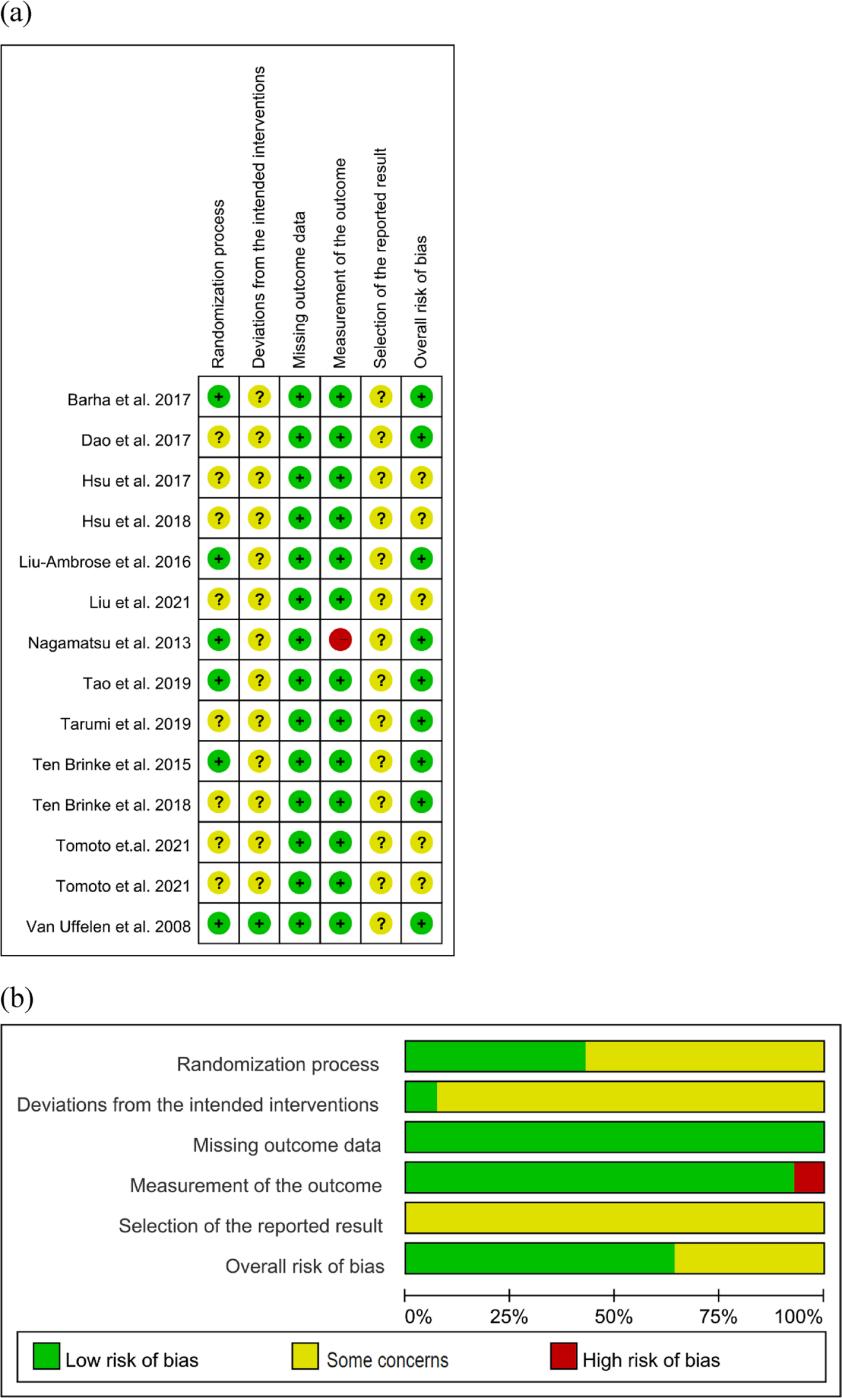
**a** Risk of bias of the included studies (*n* = 14); **b** Summary of the risk of bias. The overall risk of bias, except for blinding (performance bias), was low

The risk of bias is summarized in Fig. 2. The risk of bias due to deviations from the intended interventions and selection of the reported results of the included studies were issues of concern because we failed to obtain protocols and could not judge them. One study had a high risk of bias in the measurement of the outcomes due to uncertainty about whether the assessors were blinded.

### Effect of interventions on global cognitive function in individuals with MCI

**Fig. 3:**
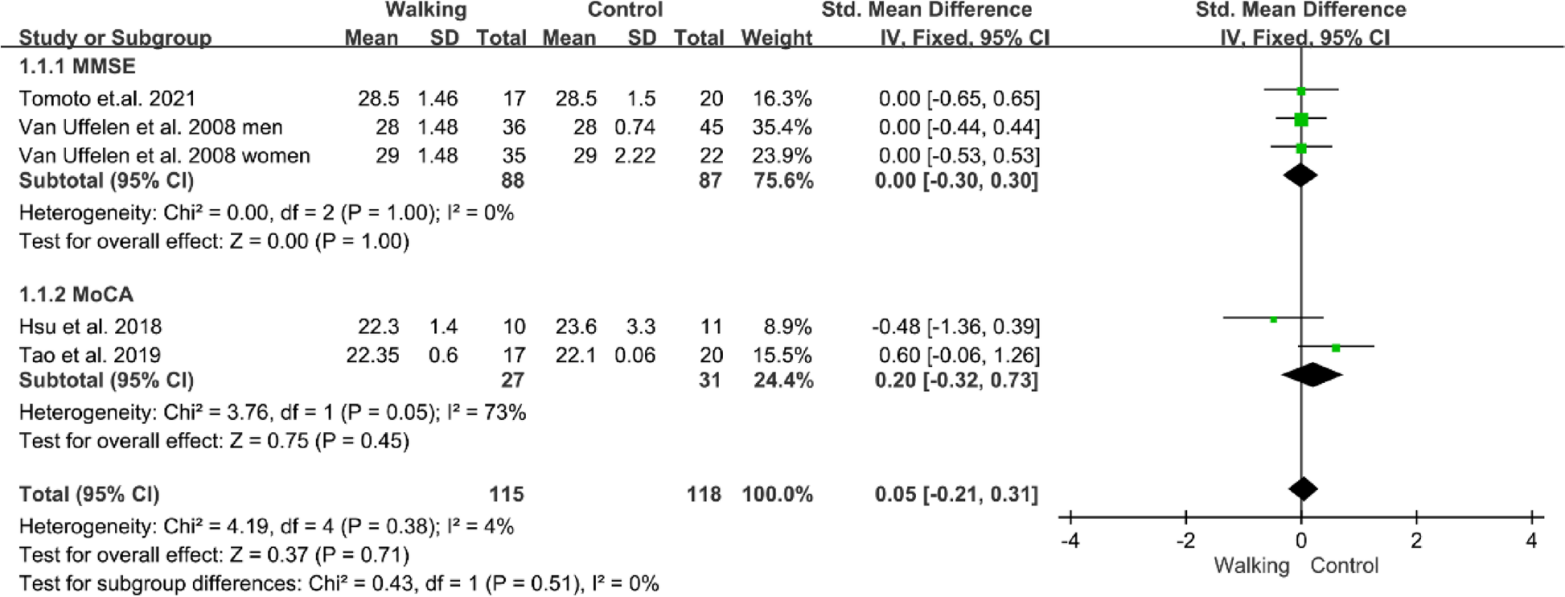
Forest plot showing the effect of walking on the MMSE and MoCA in individuals with MCI

Four studies reported the effects of walking on global cognitive function in individuals with MCI measured by MMSE and MoCA [32, 39, 41, 45]. The forest plots showed no significant difference between the walking and control groups (n=233, SMD=0.05, 95% CI: −0.21 to 0.31, *p*=0.71, *I*^2^=4%; Fig. 3).

### Effect of interventions on verbal learning in individuals with MCI

**Fig. 4:**
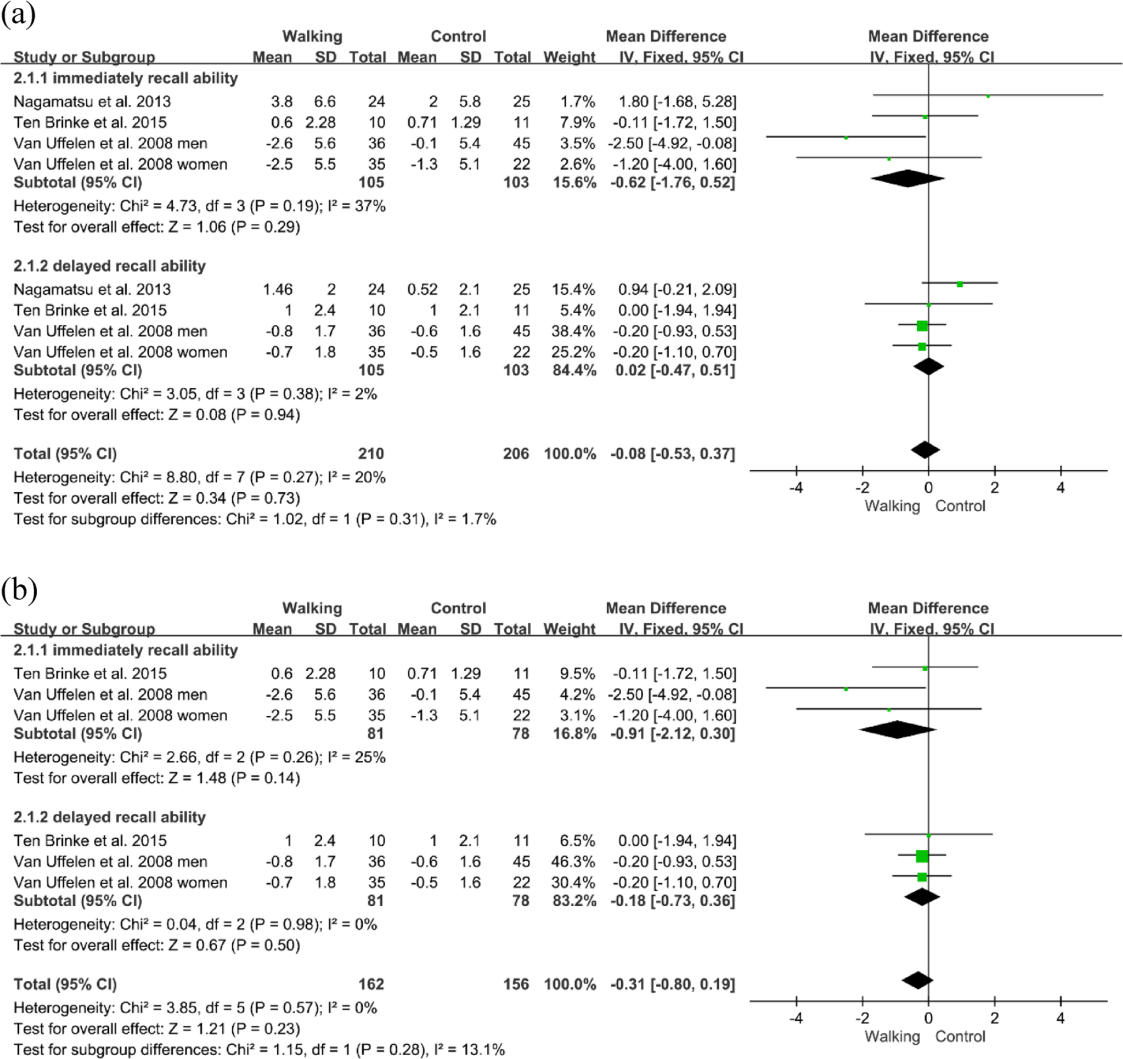
**a** Forest plot showing the effect of walking on the RAVLT in individuals with MCI; **b** Sensitivity analysis: Forest plot showing the effect of walking on the RAVLT in individuals with MCI

Three studies reported the effects of walking on verbal learning in individuals with MCI measured by RAVLT [32–34]. The forest plots showed no significant difference between the walking and control groups (n=416, MD=-0.08, 95% CI: −0.53 to 0.37, *p*=0.73, *I*^2^=20%; Fig. 4a). In sensitivity analyses, the no significant effect of walking on verbal learning remained when only studies with a low risk of bias were included (n=318, MD=-0.31, 95% CI: −0.80 to 0.19, *p*=0.23, *I*^2^=0%; Fig. 4b).

### Effect of interventions on processing speed in individuals with MCI

**Fig. 5:**
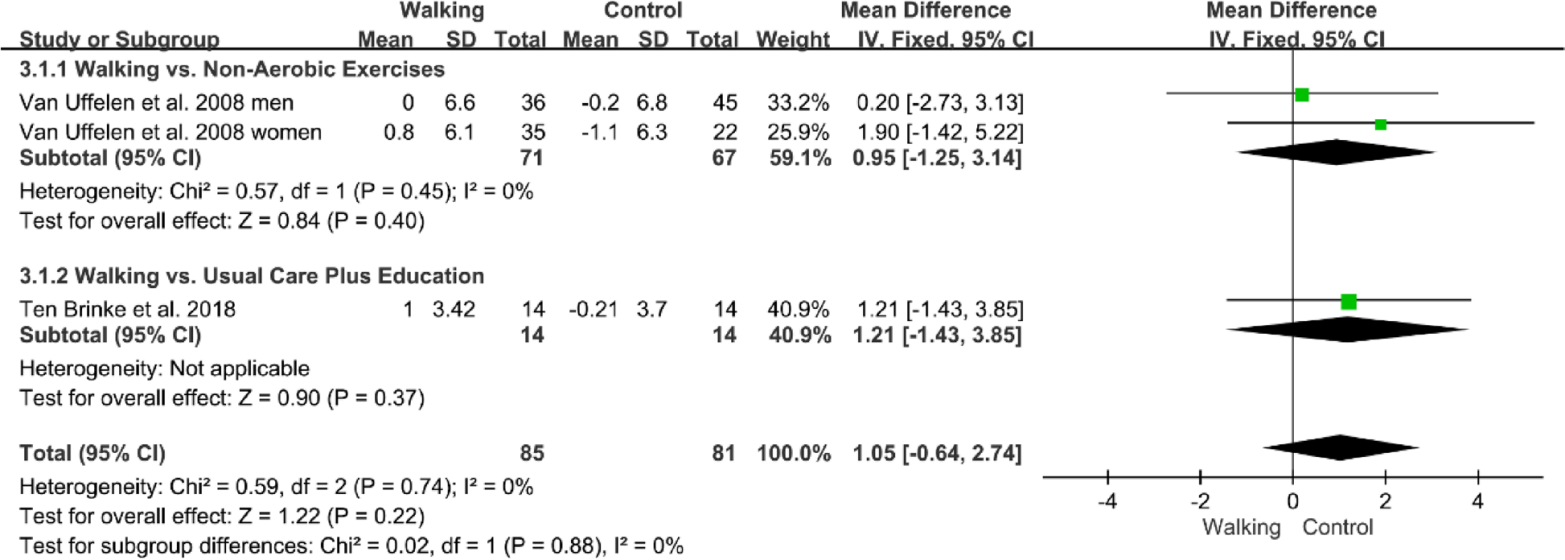
Forest plot showing the effect of walking on the DSST in individuals with MCI

Two studies reported the effects of walking on processing speed in patients with MCI measured by the DSST [32, 40]. The forest plot showed no significant difference between the walking and control groups (n=166, MD=1.05, 95% CI: −0.64 to 2.74, *p*=0.22, *I*^2^=0%; Fig. 5). No statistical difference was found between the subgroup analysis.

### Effect of interventions on walking endurance in individuals with MCI

**Fig. 6:**
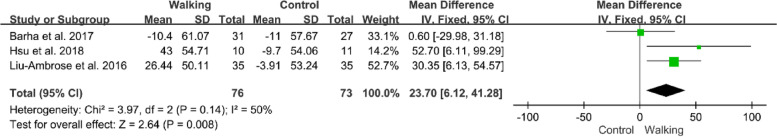
Forest plot showing the effect of walking on the 6MWT in individuals with MCI

Three studies reported the effects of walking on walking endurance in individuals with MCI measured by the 6MWT [35, 36, 38]. The forest plot showed that walking endurance was significantly improved in the experimental group compared to the control group (n=149, MD=23.70, 95% CI=6.12 to 41.28, *p*=0.008, *I*^2^=50%, Fig. 6).

### Quality of evidence: GRADE

The evidence for the effect of walking in individuals with MCI on global cognitive function comes from 4 RCTs. The RCTs suffered from inadequate concealment of allocation, no blinding of participants and exercise trainers, more than 15% of participants were lost, without using intention-to-treat analysis, and sparse data with less than 200 participants per comparison. These serious limitations would warrant downgrading the quality of evidence by two levels, from high to low. Three RCTs of the effects of walking on older adults with MCI measured verbal learning. Two studies had no blinding of participants and exercise trainers. In this case, the overall limitations were not serious and the evidence was not downgraded for risk of bias. Thus, the quality of evidence was high. The evidence for the effect of walking in individuals with MCI on processing speed comes from 2 RCTs. One study had no blinding of participants and exercise trainers and sparse data with less than 200 participants per comparison. These serious limitations would warrant downgrading the quality of evidence by one level, from high to moderate. The evidence for the effect of walking in individuals with MCI on walking endurance comes from 3 RCTs. The RCTs suffered from inadequate concealment of allocation, no blinding of participants and exercise trainers, more than 15% of participants were lost, without using intention-to-treat analysis, and sparse data with less than 200 participants per comparison. These serious limitations would warrant downgrading the quality of evidence by two levels, from high to low (Table 2).

**Table 2:** Overview of GRADE results for group comparisons concerning intervention with walking

Outcomes	Risk of bias	Inconsistency	Imprecision	Publication bias	SMD or MD (95% CI)	Number of participants (Walking vs. Control)/Studies					Quality of the evidence (GRADE)
Global cognitive function	S^a^	Ns	S^b^	Ns	SMD = -0.09 (-0.40 to 0.23)	75	78				⨁⨁◯◯ LOW
						(Four studies)					
Verbal learning	Ns	Ns	Ns	Ns	MD = -0.08 (-0.53 to 0.37)	105		103			⨁⨁⨁⨁ HIGH
						(Three studies)					
Processing Speed	Ns	Ns	S^b^	Ns	MD = 1.05 (-0.64 to 2.74)	85				81	⨁⨁⨁◯ MODERATE
						(Two studies)					
Walking endurance	S^a^	Ns	S^b^	Ns	MD = 23.70 (6.12 to 41.28)	76			73		⨁⨁◯◯ LOW
						(Three studies)					

(Ns) No serious; (S) Serious

^a^Downgrading due to < 75% of the studies presented high quality

^b^Downgrading due to sparse data with less than 200 participants per comparison

## Discussion

This systematic review and meta-analysis aimed to evaluate the effects of walking on cognitive functions and walking endurance in individuals with MCI. The present meta-analysis found that walking intervention compared to no intervention or other exercises cannot significantly improve cognitive function in patients with MCI. However, significant improvement in walking endurance was observed in the walking groups compared to the control groups that received no intervention or other exercises.

In this meta-analysis, we analyzed the results of 14 RCT studies and demonstrated that walking interventions may not have significant effects on global cognition or specific domains of cognition tests in people with MCI. Demurtas et al. investigated the cognitive benefits of physical activity and exercise in older adults with cognitive impairment in an umbrella review [46]. Twenty-seven systematic reviews (all RCTs) were included, and the results showed that physical activity/exercise is able to significantly improve global cognition and specific cognitive functions. Another meta-analysis including 13 RCTs reported that aerobic exercise training produces small improvements in executive function among older adults [47]. The possible explanations why our findings were inconsistent with previous studies might be due to the exercise intensity. A previous meta-analysis indicated that high frequency (more than 5 times per week) and moderate-to-vigorous intensity exercise yielded positive improvements in cognitive function [47] with the largest effect size, followed by Tai Chi and yoga, resistance exercise, combined exercise, and aerobic exercise. Mavros et al. noted that the high-intensity progressive resistance training intervention for MCI shows a significant improvement in global cognition [48]. Thomas et al. conducted a study in older adults with MCI, implementing a treadmill walking intervention ranging from moderate to high intensity. The results showed a significant improvement in language learning and a significant increase in anterior cingulate cortex cerebral blood flow [49]. Another possible explanation why walking could not have positive effects on cognition might be because walking is a relatively simple exercise that has less impact on cognitive functions. De Oliveira Silva et al. demonstrated that 12 weeks of moderate-intensity multimodal training (aerobic, strength, balance, and flexibility) contributes to significant improvements in executive function in older adults with MCI [50]. A cohort study followed 469 dementia-free older adults for five years and found that dance was the only exercise activity, compared to walking, stair climbing, biking, and swimming, to significantly reduce the risk of dementia [51]. A systematic review and meta-analysis including 13 studies reported that dance had positive effects on global cognition and memory in older adults [52]. Although walking is an aerobic exercise that can be performed every day, its exercise intensity is relatively low and the exercise type is relatively simple, which may result in a poor effect on cognitive function in older adults with MCI.

Although our meta-analysis results showed that a walking intervention compared to no intervention or other exercises cannot significantly improve cognition tests in people with MCI, walking may still have positive effects on brain health. Two of our included articles indicated that walking intervention can improve cerebrovascular function, enhance cerebral perfusion and increase cerebral blood flow in older adults with MCI [43, 45]. Another included study reported that the walking exercise of people with MCI showed no significant improvement in MoCA but had increased effective connectivity from the right anterior cingulate cortex to the left ventral tegmental area and the right locus coeruleus through magnetic resonance imaging (MRI) [44]. Progressive walking programs increase the hippocampal volume [34, 53], and increased hippocampal volume is associated with greater levels of plasma brain-derived neurotrophic factor [53]. A previous study reported that higher levels of physical activity measured by a pedometer were linked to better white matter health in aging [54]. These results indicate that the amount of improvement from walking intervention may be not sufficient to observe in clinical cognitive tests, but brain changes followed by walking can be measured using precise assessment tools such as MRI.

In addition, the results of this meta-analysis suggest that walking intervention has a positive effect on the 6MWT in older adults with MCI. A previous meta-analysis reported that RCTs of exercise training resulted in moderate to large positive effects on the 6MWT in individuals with MCI [55], which was consistent with our findings. The 6MWT has been proposed as a valuable tool to assess aerobic capacity and endurance [56], and performance on the 6MWT is independently related to all-cause mortality in older adults [57]. Therefore, we hypothesize that people with MCI can benefit from walking intervention to improve walking endurance, which may decrease mortality in older adults with MCI.

### Study limitations and clinical implications

There are some limitations that need to be addressed when interpreting the current results. First, there was considerable heterogeneity in the included studies regarding the diagnosis (e.g., Petersen criteria, DSM-5, MMSE ≥ 24/30 and MoCA < 26/30), types of MCI (e.g., amnestic MCI, non-amnestic MCI) and intervention characteristics (e.g., intervention period, frequency, duration). Clinical heterogeneity may have influenced the findings; however, the small number of eligible studies included in this meta-analysis prevented us from performing further subgroup analysis. Second, we only included articles that were published in English, which may have resulted in a language bias. Besides, we could not obtain information on publication bias because every test for funnel plot asymmetry can be used only when there are at least 10 studies included in the meta-analysis. When there are fewer studies, the power of the tests is too low to distinguish chance from real asymmetry [58]. Finally, it should be noted that some of the included literature in this review comes from the same research team, which may introduce a risk of bias.

Although walking is the most common type of exercise performed by community-dwelling older adults, the current meta-analysis does not provide evidence to support a greater effect when walking is compared to no intervention or other exercises in individuals with MCI. However, walking has beneficial effects on aerobic capacity and brain health. Therefore, due to the lower costs of walking intervention compared to supervised exercise programs [59], walking plays a valuable role in the maintenance of physical health in MCI.

## Conclusion

The results of the present meta-analysis report that walking intervention does not improve global cognitive function and specific domains of cognition when compared to no intervention or other exercises in older adults with MCI. Nevertheless, walking induces beneficial effects on aerobic capacity. Future studies should investigate the effects of different walking dosages and precise intervention programs for individuals with different types of MCI.

### Supplementary Information

**Additional file 1: Appendix 1.** Retrieval strategy.

## Data Availability

The datasets used and/or analyzed during the current study are available from the corresponding author on reasonable request.

## Abbreviation

MCI: Mild Cognitive Impairment
RoB 2: Revised Cochrane Risk of Bias assessment tool
PRISMA: Preferred Reporting Items for Systematic Reviews and Meta-Analyses
GRAGE: Grading of Recommendations, Assessment, Development and Evaluations
RCT: Randomized Controlled Trials
DSM-5: US mental disorders fifth edition of the Diagnostic and Statistical Manual
MMSE: Mini-Mental State Examination
MoCA: Montreal Cognitive Assessment
RAVLT: Rey Auditory Verbal Learning Test
DSST: Digit Symbol Substitution Test
6MWT: 6-Minute Walk Test
MRI: Magnetic Resonance Imaging
