# Supplementary material for: Review articles (Meta-Analyses) effects of walking on cognitive function in individuals with mild cognitive impairment: a systematic review and meta-analysis
Source: BMC Geriatr. 2023 Aug 21;23:500. doi: 10.1186/s12877-023-04235-z (PMC10441758; doi:10.1186/s12877-023-04235-z)
Supplement: Supplementary file 1 — Additional file 1: Appendix 1. Retrieval strategy. [file 12877_2023_4235_MOESM1_ESM.docx]

Appendix 1. Retrieval strategy

| ID Search Hits | Result |
| --- | --- |
| MEDLINE |  |
| 1. (MH "Cognitive Dysfunction") | 35735 |
| 1. (MH "Cognitive Dysfunction") OR Mild Cognitive Impairment OR Mild Neurocognitive Disorders | 49576 |
| 1. (MH "Walking") | 41341 |
| 1. (MH "Walking") OR Walking OR Walking Exercise OR Brisk Walking | 104094 |
| 1. #2 AND #4 | 502 |
|  |  |
| PubMed |  |
| 1. "cognitive dysfunction"[MeSH Terms] OR ("cognitive dysfunction"[MeSH Terms] OR ("cognitive"[All Fields] AND "dysfunction"[All Fields]) OR "cognitive dysfunction"[All Fields] OR ("mild"[All Fields] AND "cognitive"[All Fields] AND "impairment"[All Fields]) OR "mild cognitive impairment"[All Fields]) OR ("neurocognitive disorders"[MeSH Terms] OR ("neurocognitive"[All Fields] AND "disorders"[All Fields]) OR "neurocognitive disorders"[All Fields] OR ("mild"[All Fields] AND "neurocognitive"[All Fields] AND "disorders"[All Fields]) OR "mild neurocognitive disorders"[All Fields]) | 349889 |
| 1. "walking"[MeSH Terms] OR ("walked"[All Fields] OR "walking"[MeSH Terms] OR "walking"[All Fields] OR "walks"[All Fields]) OR (("walked"[All Fields] OR "walking"[MeSH Terms] OR "walking"[All Fields] OR "walks"[All Fields]) AND ("exercise"[MeSH Terms] OR "exercise"[All Fields] OR "exercises"[All Fields] OR "exercise therapy"[MeSH Terms] OR ("exercise"[All Fields] AND "therapy"[All Fields]) OR "exercise therapy"[All Fields] OR "exercising"[All Fields] OR "exercise s"[All Fields] OR "exercised"[All Fields] OR "exerciser"[All Fields] OR "exercisers"[All Fields])) OR ("Brisk"[All Fields] AND ("walked"[All Fields] OR "walking"[MeSH Terms] OR "walking"[All Fields] OR "walks"[All Fields])) | 129418 |
| 1. #1 AND #2 | 2803 |
| 1. (#1) AND (#2) Filters: Randomized Controlled Trial | 203 |
|  |  |
| SPORTDiscus |  |
| 1. Mild Cognitive Impairment OR Mild Neurocognitive Disorders OR Cognitive Dysfunction | 836 |
| 1. walking OR Walking Exercise OR Brisk Walking OR Exercise | 187159 |
| 1. #1 AND #2 | 169 |
|  |  |
| Cochrane Central Register of Controlled Trials |  |
| 1. MeSH descriptor: [Cognitive Dysfunction] explode all trees | 3023 |
| 1. (Mild Cognitive Impairment):ti,ab,kw OR (Mild Neurocognitive Disorders):ti,ab,kw | 6133 |
| 1. #1 or #2 | 7960 |
| 1. MeSH descriptor: [Walking] explode all trees | 8487 |
| 1. (Walking):ti,ab,kw OR (Walking Exercise):ti,ab,kw OR (Brisk Walking):ti,ab,kw | 40592 |
| 1. #4 or #5 | 41726 |
| 1. #3 and #6 | 461 |
|  |  |
| CINAHL |  |
| 1. (MH "Mild Cognitive Impairment") | 885 |
| 1. (MH "Mild Cognitive Impairment") OR Mild Cognitive Impairment OR Mild Neurocognitive Disorders | 10624 |
| 1. (MH "Walking") | 25211 |
| 1. (MH "Walking") OR Walking Exercise OR Brisk Walking | 26520 |
| 1. #2 and #4 | 52 |
|  |  |
| Web of Science |  |
| 1. ((ALL=(Mild Cognitive Impairment)) OR ALL=(Mild Neurocognitive Disorders)) OR ALL=(Cognitive Dysfunction ) | 109091 |
| 1. (((ALL=(Walking )) OR ALL=(Walking Exercise )) OR ALL=(Brisk Walking )) OR ALL=(Exercise) | 823061 |
| 1. #1 AND #2 | 4755 |
|  |  |
| Airiti Library |  |
| 1. (([ALL]:(Mild Cognitive Impairment) OR [ALL]:(Mild Neurocognitive Disorders)) OR [ALL]:(Cognitive Dysfunction )) | 9044 |
| 1. ((([ALL]:(Walking) OR [ALL]:(Walking Exercise)) OR [ALL]:(Brisk Walking)) OR [ALL]:(Exercise)) | 111822 |
| 1. #1 AND #2 | 177 |
|  |  |
| The National Digital Library of Theses and Dissertations in Taiwan |  |
| 1. "Mild Cognitive Impairment" or "Mild Neurocognitive Disorders" or "Cognitive Dysfunction" | 2693 |
| 1. "Walking" or "Walking Exercise" or "Brisk Walking" or "Exercise" | 59294 |
| 1. (#1) and (#2) | 926 |
